# TgAP2X-7 is a novel cell cycle-regulated transcription factor that plays an essential role in *Toxoplasma* tachyzoite propagation

**DOI:** 10.1128/msphere.00438-25

**Published:** 2025-09-08

**Authors:** Padmaja Mandadi, Ramu Anandakrishnan, Rajshekhar Y. Gaji

**Affiliations:** 1 Department of Biomedical Sciences and Pathobiology, Virginia-Maryland College of Veterinary Medicine 229659, Blacksburg, Virginia, USA; 2 Department of Biomedical Sciences, Edward Via College of Osteopathic Medicine (VCOM) 41066, Blacksburg, Virginia, USA; Australian National University, Canberra, Australia

**Keywords:** parasitology, apicomplexan parasites, *Toxoplasma gondii*, transcription factors

## Abstract

Apicomplexan AP2 (ApiAP2) family proteins are a family of transcription factors that are known to regulate gene expression in apicomplexan pathogens, including *Toxoplasma*. In this study, we focused on TgAP2X-7, a member of the APiAP2 family that is predicted to be essential for *Toxoplasma* fitness. Endogenous tagging of TgAP2X-7 followed by immunofluorescence analysis revealed that it’s a cell cycle-regulated nuclear protein with peak expression in the G1 phase. Since TgAP2X-7 is predicted to be essential for parasite growth, we adopted an auxin-inducible degron (AID) based conditional knockdown approach to understand its function. Phenotypic analysis of the conditional knockdown mutant of TgAP2X-7 showed that the protein is indeed essential for *Toxoplasma* propagation *in vitro,* and loss of this transcription factor results in a major defect in invasion and a minor defect in replication. Examination with cell division markers indicated that the absence of TgAP2X-7 results in defects in endodyogeny. Transcriptomic analysis indicated that loss of TgAP2X-7 leads to dysregulation of global gene expression in the parasite, including genes required for host-cell invasion, metabolism, and gene expression. Additionally, Cleavage Under Targets and Tagmentation (CUT&TAG) analysis suggests that TgAP2X-7 likely binds to an 11 bp motif [C/T/G]GCATGCA[G/C/A][C/T/G][G/A] in the parasite genes’ promoter region. Together, these findings suggest that TgAP2X-7 is a novel transcriptional regulator in *Toxoplasma* that governs the expression of genes required for parasite propagation.

**IMPORTANCE:**

*Toxoplasma gondii* is a protozoan parasite that can cause life-threatening disease in mammals; hence, identifying key factors required for parasite growth and pathogenesis is important to develop novel therapeutics. In this study, we identify and characterize a member of the Apicomplexan AP2 (ApiAP2) family, TgAP2X-7, a developmentally regulated transcription factor. By generating conditional mutant TgAP2X-7, we show that this protein is required for *Toxoplasma* propagation *in vitro,* and the absence of this protein results in parasites with significantly reduced competency in invasion, moderate deficiency in replication, and defects in cell division. Importantly, TgAP2X-7-deficient parasites show global changes in gene expression profile, including decreased expression of genes important for *Toxoplasma* entry into the host cell. Additionally, we identified an 11 bp DNA motif likely recognized by this transcription factor. Hence, this study provides an initial insight into the function of a novel cell cycle-regulated transcription factor essential for *Toxoplasma* growth.

## INTRODUCTION

*Toxoplasma gondii* is an obligatory intracellular protozoan parasite classified in the phylum Apicomplexa (1). The phylum includes important human and animal pathogens, including *Plasmodium,* which causes malaria, *Cryptosporidium* that causes diarrhea in neonates, *Neospora caninum* that causes abortion in cattle, and *Sarcocystis neurona* that causes neurological disease in horses (2–6).

*Toxoplasma* follows an indirect mode of life cycle with the feline species (mainly cat) as the definitive host and a large number of farm animals and birds serving as the intermediate hosts (1). The sexual phase of the *Toxoplasma* life cycle occurs in the intestinal epithelial cells of the definitive host and is shed in feces as oocysts. The intermediate hosts pick up *Toxoplasma* infection through ingestion of food or water contaminated with oocysts. Humans, however, acquire *Toxoplasma* infection mainly in three ways: ingestion of food or water contaminated with *Toxoplasma* oocysts, ingestion of contaminated meat that contains parasite cysts, and transplacental transmission during pregnancy (1, 7).

Following infection with *Toxoplasma*, the acute form of the parasite (tachyzoite) disseminates quickly into various organs in the host. In response to the subsequent host immune response, the parasite evades elimination by converting into an encysted form (bradyzoites) and establishing a chronic infection that persists through the life of the host (2). In immunocompromised individuals, such as those infected with HIV, and those undergoing immunosuppressive therapy during organ transplantation, reactivation of the chronic infection or the establishment of new acute infections can lead to fatal toxoplasmosis (8–10). Additionally, transmission from the mother to the fetus during pregnancy results in congenital toxoplasmosis, causing severe disease in the offspring, including neurological deficiencies and blindness, sometimes resulting in neonatal death (7, 11). Despite being an important human pathogen, currently, there is no vaccine to prevent *Toxoplasma* infection, and the drugs currently used have severe side effects (2). As a result, there is a critical need for the identification and development of novel therapeutic options to treat *Toxoplasma* infections.

The intracellular lifestyle of *Toxoplasma* begins with the parasite gaining entry into a host cell through an active invasion process (12, 13). Once in the host cell, the parasite resides within a parasitophorous vacuole and replicates through a process known as endodyogeny (14–17). After undergoing multiple rounds of division, the daughter parasites egress from the host cell, which is destroyed in the process (18, 19). Importantly, much of the pathology associated with *Toxoplasma* infection is due to the extensive tissue destruction caused by repeated cycles of invasion, replication, and egress in the infected host (16, 19–21). Consequently, identifying unique transcription factors that regulate the expression of proteins involved in these lytic cycle events could open new opportunities for therapeutic interventions against *Toxoplasma* (16, 19).

Transcriptional regulation of gene expression in *Toxoplasma* appears to be mediated through a unique set of factors, namely, Apicomplexan AP2 (ApiAP2) family proteins (22–25). These ApiAP2 proteins are homologous to plant Apetela2/Ethylene Response Factor (AP2/ERF) transcription factors, first described in *Arabidopsis* (26). These ApiAP2 proteins contain AP2 domains that have been shown to bind specific motifs on the DNA and thus regulate gene expression in apicomplexan parasites (27). *Toxoplasma* contains 67 members of the ApiAP2 family in its genome, and of these, 28 proteins show constitutive expression while 32 members show cell cycle regulation during endodyogeny. Many of the AP2 proteins have been shown to play a role in the progression of cell division in *Toxoplasma*, e.g., VIIa-4, IX-7, and XII-4 are involved in G1-S transition, while IV-4, XI-1, and XII-9 are important for daughter cell budding. Although we are now beginning to understand the role of some APiAP2 family members in *Toxoplasma*, the functional significance of a large number of AP2 factors still remains undefined (23, 28).

In this study, we focused on TgAP2X-7, an uncharacterized member of the ApiAP2 family in *Toxoplasma* that was suggested to be important for parasite growth *in vitro* (29). Our findings reveal that TgAP2X-7 is a cell cycle-regulated protein that localizes to the parasite nucleus. Since TgAP2X-7 was predicted to be essential for *Toxoplasma* viability, we generated a conditional knockdown mutant to determine its function. Our findings reveal that TgAP2X-7 is indeed essential for parasite propagation, as the absence of this protein results in loss of plaque formation. Further dissection of the parasite lytic cycle revealed that TgAP2X-7-deficient parasites show severe defects in invasion and moderate defects in replication. Importantly, RNA-seq analysis indicated that TgAP2X-7 is involved in the regulation of a large gene repertoire in *Toxoplasma*. Interestingly, Cleavage Under Targets and Tagmentation (CUT&TAG) analysis showed this protein likely binds to an 11 bp DNA motif. Together, these results establish that TgAP2X-7 is a novel cell cycle-regulated transcription factor that plays a key role in the *Toxoplasma* lytic cycle and pathogenesis.

## RESULTS

### TgAP2X-7 is a cell cycle-regulated transcription factor in *Toxoplasma*

**Fig 1:**
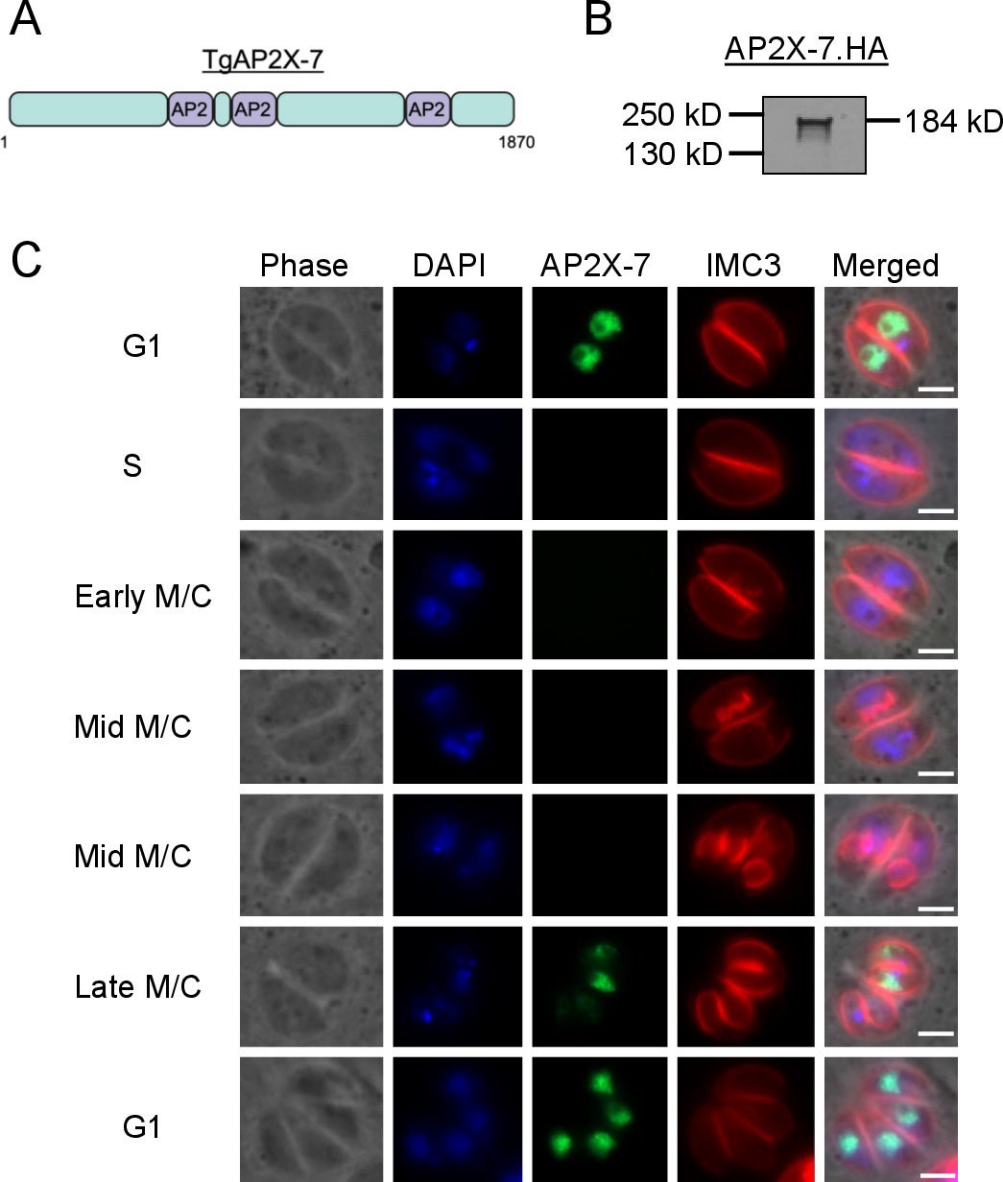
TgAP2X-7 is a cell cycle-regulated nuclear protein. (**A**) Schematic representation of TgAP2X-7 protein. The location of AP2 domains on the protein is indicated. (**B**) Western blot analysis of TgAP2X-7-HA parasites with anti-HA antibody. (**C**) Localization of TgAP2X-7 in intracellular parasites at different stages of the cell cycle using anti-HA antibody. IMC3, which localizes to the pellicle, is used as a marker for different cell cycle stages. DAPI stains the nucleus. Scale bar, 2 µm.

TgAP2X-7 is a member of the ApiAP2 family, which is 1,876 amino acids in length and contains three AP2 domains (Fig. 1A). To determine the localization of TgAP2X-7 in *Toxoplasma*, we introduced an HA epitope tag at the 3′ end of the endogenous gene (30). Western blot analysis using an anti-HA antibody revealed a single band of the expected size in the endogenously HA-tagged clone (Fig. 1B). A previous cell cycle transcriptome analysis suggested that TgAP2X-7 is a cell cycle-regulated transcription factor (31). Immunofluorescence analysis using anti-HA antibody showed that the TgAP2X-7 localizes to the parasite nucleus and its expression is indeed temporally regulated. Specifically, TgAP2X-7 is expressed in the G1 stage of the cell cycle, and the protein disappears as the parasites advance to the S phase. Furthermore, the protein continues to be absent in the early and mid-mitosis/cytokinesis (M/C) phases but shows weak expression in late M/C stages. As the parasites complete cell division and return to the G1 phase, a strong signal is noticed for TgAP2X-7 (Fig. 1C). We also examined the expression of TgAP2X-7 using the centrosomal marker, TgCentrin1, and early daughter cell budding marker, inner membrane complex (IMC) sub-compartment protein 1 (ISP1), a protein that localizes to the apical end of the IMC. The results showed that TgAP2X-7 is present in parasites with an undivided centrosome, absent in parasites with divided centrioles, and reappears as the daughter parasites return to G1 phase after division (Fig. S1A). Similarly, the results using ISP1 revealed that TgAP2X-7 is not expressed when daughter buds are forming, but the transcription factor makes an appearance after the cell division is complete (Fig. S1B). Together, these findings suggest that TgAP2X-7 is a cell cycle-regulated protein in *Toxoplasma*, with peak expression in the G1 phase, weak expression in the late M/C phase, and complete absence of expression in the S phase.

### Generation of conditional knockdown strain of TgAP2X-7

Next, we wanted to determine the role of TgAP2X-7 in *Toxoplasma* propagation. Since a previous study that conducted genome-wide analysis to identify genes important for *Toxoplasma* fitness suggested that TgAP2X-7 is an essential gene (29), we wanted to generate a conditional knockdown mutant of this gene. Toward this goal, we used the recently developed auxin-inducible degron (AID) system (32). This approach involves insertion of a C-terminal tag of mAID domain followed by HA epitope at the 3′ end of TgAP2X-7 gene in the parasite line stably expressing auxin receptor, TIR1 (32). The protein with the mAID tag will function normally in the parasites in the absence of auxin. However, when auxin is added to these parasites, the mAID tag will result in ubiquitination followed by proteasomal degradation of the tagged protein.

**Fig 2:**
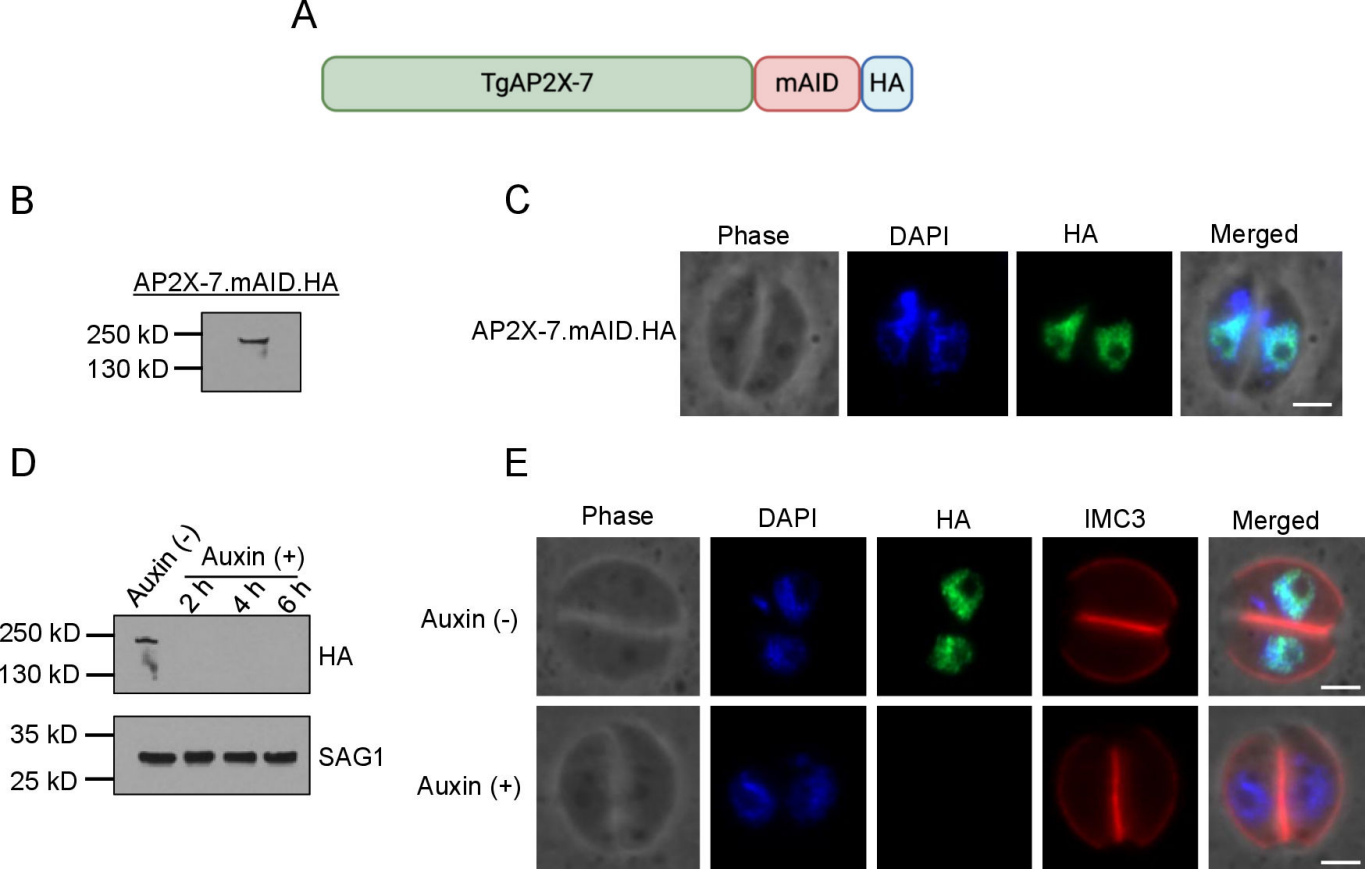
Generation of conditional knockdown strain of TgAP2X-7 using the AID system. (**A**) Schematic representation of mAID-HA tagged TgAP2X-7 protein. (**B**) Western blot analysis of TgAP2X-7-mAID-HA strain using anti-HA antibody. (**C**) TgAP2X-7.mAID.HA protein shows nuclear localization. DAPI stains the nucleus. (**D**) Immunoblot analysis of TgAP2X-7.mAID.HA strain using anti-HA body treated with vehicle control or auxin. SAG1 is used as a loading control. (**E**) Immunofluorescence assay confirming degradation of TgAP2X-7 after treatment with auxin (IAA or indole-3-acetic acid). TgAP2X-7 was visualized using an anti-HA antibody, and the inner membrane complex was stained with IMC3.

Accordingly, we introduced the mAID-HA tag at the C terminus of TgAP2X-7 by CRISPR-Cas9 technology to generate the TgAP2X-7.mAID.HA strain as described in Materials and Methods (Fig. 2A). Immunoblotting using anti-HA antibody revealed a single band of expected size for TgAP2X-7 protein (Fig. 2B). Immunofluorescence assay (IFA) analysis showed that TgAP2X-7.mAID.HA localizes to the parasite nucleus as observed previously with just the HA epitope tag (Fig. 2C). Furthermore, downregulation of TgAP2X-7 was examined by immunoblotting after treating with vehicle control or with auxin, and the results showed that the protein completely disappears as early as 2 h in the presence of auxin (Fig. 2D). We also performed IFA analysis of TgAP2X-7.mAID.HA strain using anti-HA antibody. The results revealed a complete absence of TgAP2X-7 protein in the parasite nucleus treated with auxin (Fig. 2E), thus suggesting successful establishment of a conditional knockdown strain of TgAP2X-7 protein.

### TgAP2X-7 is essential for *Toxoplasma* propagation *in vitro*

**Fig 3:**
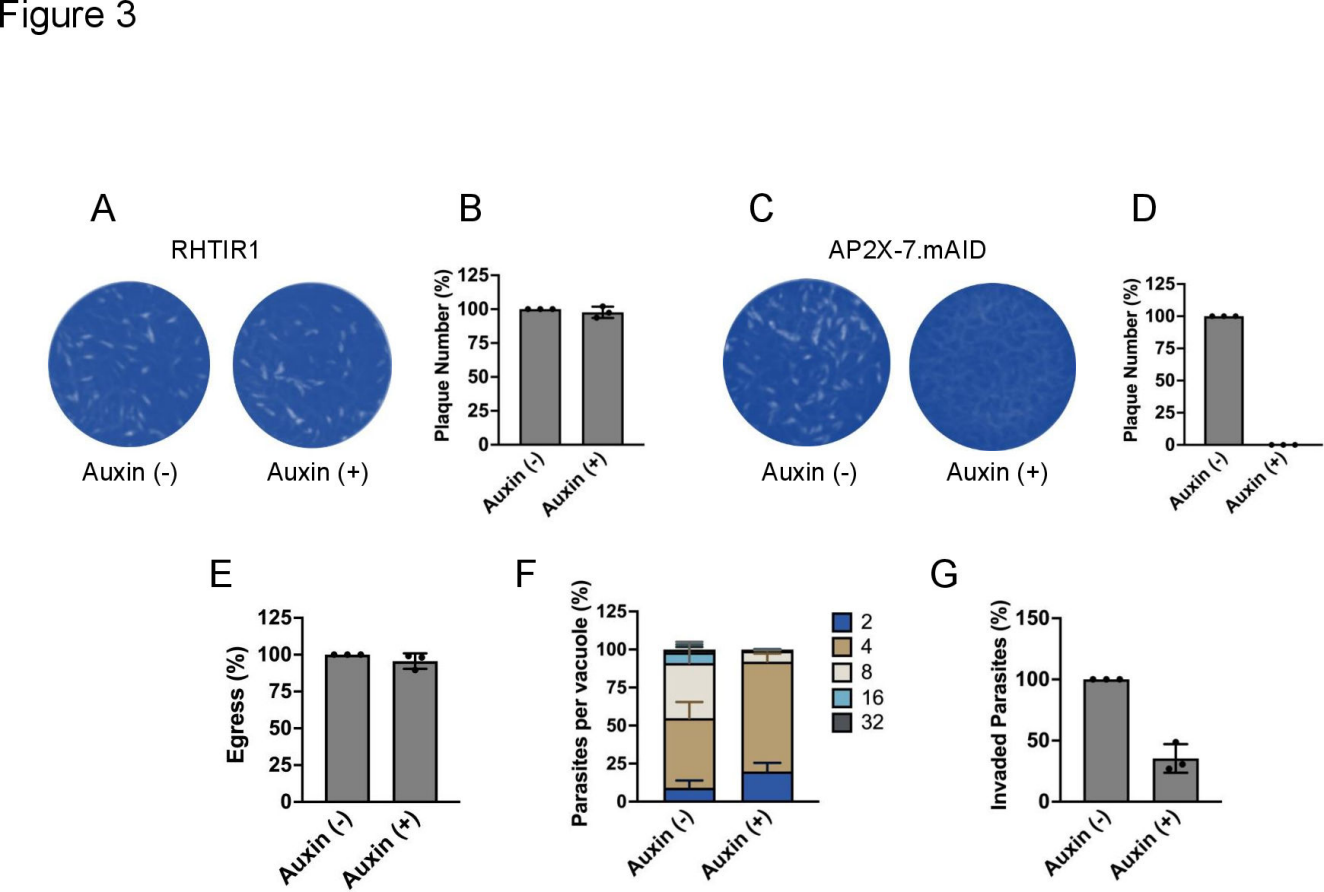
Loss of TgAP2X-7 results in the abolition of Toxoplasma growth in vitro and significantly impairs host cell invasion. (**A**) Plaque assays were performed to examine the growth of parental strain (RH.TIR1) in the presence or absence of auxin. Plaques are visible as clear zones on the background of crystal violet stained HFF monolayer. (**B**) Quantification of plaque numbers of RH.TIR1 strain grown in the presence or absence of auxin. *n* = 3, data represent mean ± standard deviation. (**C**) Plaque assays were performed to examine the growth of TgAP2X-7-mAID-HA strain in the presence or absence of auxin. Plaques are visible as clear zones on the background of crystal violet stained HFF monolayer. (**D**) Quantification of plaque numbers of TgAP2X-7-mAID-HA strain grown in the presence or absence of auxin. *n* = 3, data represent mean ± standard deviation. *P < 0.001, Students’ *t*-test (GraphPad Prism). (**E**) Ionophore induced egress assay of TgAP2X-7-mAID-HA strain performed in presence or absence of auxin. *n* = 3, data represent mean ± standard deviation. *P < 0.05, ns = not significant, Students’ *t*-test (GraphPad Prism). (**F**) Replication assay of TgAP2X-7-mAID-HA strain performed in presence or absence of auxin. n=3, data represent mean ± standard deviation. *P < 0.05, ns = not significant, Students’ *t*-test (GraphPad Prism). (**G**) Host-cell invasion assay of TgAP2X-7-mAID-HA parasites (treated with or without auxin) after 30 min of incubation with HFF cells. Data represent mean ±standard deviation, *n* = 3 biological replicates. ***P < 0.001, Students’ *t*-test (GraphPad Prism).

Next, we wanted to assess the importance of TgAP2X-7 in parasite growth *in vitro* by monitoring the formation of plaques on a confluent human foreskin fibroblast (HFF) monolayer that results from multiple rounds of parasite invasion, replication, and egress. Since the downregulation of TgAP2X-7 involves culturing parasites in the presence of auxin, we first wanted to test if auxin treatment has any adverse effect on *Toxoplasma* growth. Toward this goal, we performed plaque assays for the parental strain RH.TIR1 in the presence or absence of auxin. The results showed that there was no significant difference in the number of plaques formed by the RH.TIR1 strain in the presence or absence of auxin (Fig. 3A and B). These findings suggested that auxin treatment does not affect *Toxoplasma* propagation *in vitro*. Next, to determine the contribution of TgAP2X-7 to parasite growth, plaque assays were performed using the TgAP2X-7.mAID.HA strain. The results revealed that while plaques were seen in wells without auxin, parasites failed to form any plaques in the presence of auxin (Fig. 3C). Hence, results compiled from three independent experiments showed 0% plaques in auxin-treated wells (Fig. 3D), thus suggesting that TgAP2X-7 protein is essential for *Toxoplasma* propagation *in vitro*.

### TgAP2X-7-deficient parasites show defects in host-cell invasion and parasite replication

An impairment of plaque formation can be caused by defects in one or more steps of the parasite lytic cycle (19). Therefore, we next sought to determine which aspect of the lytic cycle was affected in the absence of TgAP2X-7. We first performed ionophore-induced egress assays using the Ca^2+^ ionophore A23187 (33, 34). However, we did not see significant differences in the egress capability of parasites in the presence or absence of auxin (Fig. 3F), suggesting that TgAP2X-7 does not play a role in *Toxoplasma* egress from the host cells. We next assessed parasite replication using standard doubling assays (23), and we observed that parasites treated with auxin contained fewer parasites per vacuole compared to non-treated parasites, thus suggesting that TgAP2X-7 is required for efficient intracellular growth of *Toxoplasma* (Fig. 3G). Finally, we conducted a parasite invasion assay into host cells (35), and the results revealed that there is a significant reduction (65%) in the number of invaded parasites in the auxin-treated strain compared to untreated parasites, suggesting that TgAP2X-7 is a critical determinant for *Toxoplasma* invasion (Fig. 3H).

### Loss of TgAP2X-7 results in death of intracellular *Toxoplasma*

**Fig 4:**
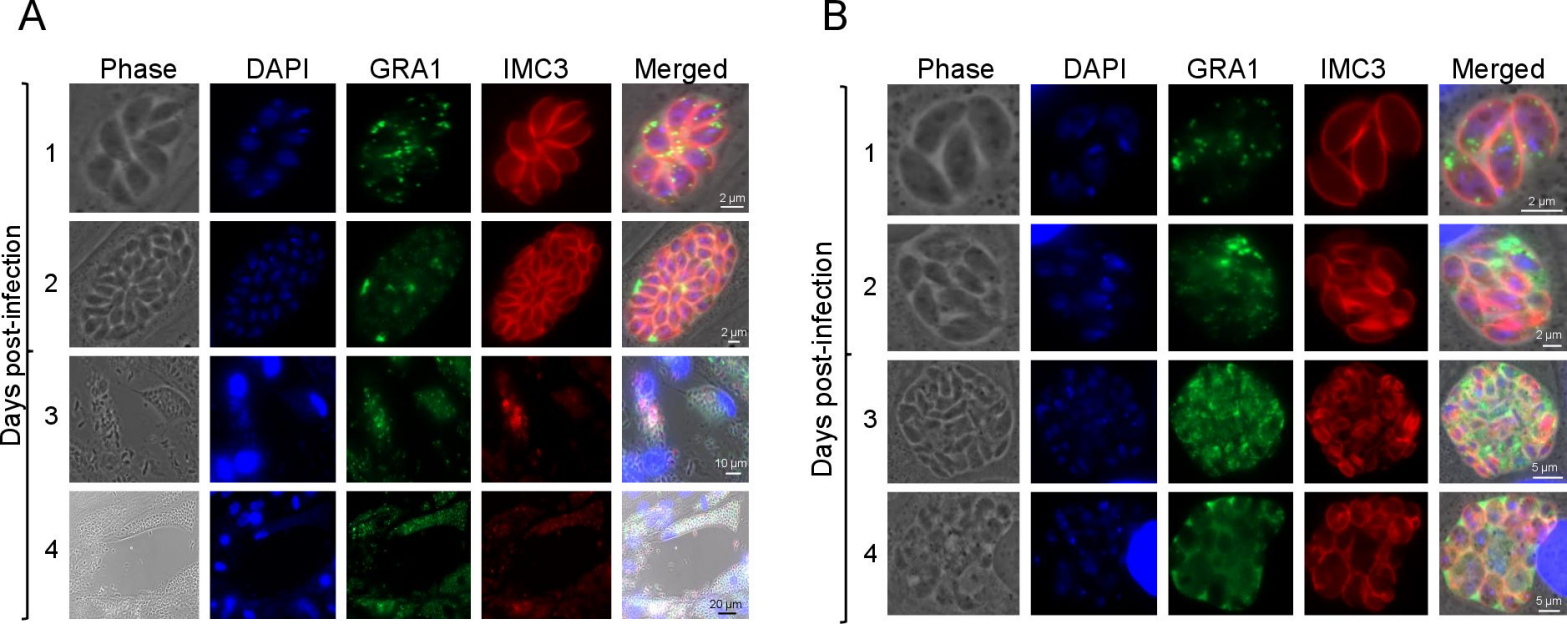
Loss of TgAP2X-7 abolishes *Toxoplasma* propagation *in vitro*. TgAP2X-7-mAID-HA parasites were added onto coverslips in 24-well plates, and immunofluorescence assays (IFA) were performed every day for 5 days starting 24 h post-infection. Intracellular parasites were stained with anti-HA antibody and anti-IMC3 antibodies to visualize TgAP2X-7 protein and IMC, respectively. DAPI stains the nucleus. Scale bars are indicated. Panel (**A**) shows the TgAP2X-7-mAID-HA strain grown without auxin, and panel (**B**) shows the TgAP2X-7-mAID-HA strain cultured in the presence of auxin.

The plaque assays suggested that TgAP2X-7 is essential for parasite propagation. The results from the lytic cycle assays indicated that the lack of TgAP2X-7 leads to moderate defects in replication and severe defects in host-cell invasion. However, since we do see a lower percentage of parasites still able to invade host cells, one can expect to see some plaque formation, but not a complete lack of growth in parasites lacking TgAP2X-7. To determine a reasonable explanation for the complete lack of plaque formation, we wanted to examine the parasite growth within the host cells. Hence, we cultured parasites in the presence or absence of auxin and fixed and stained them every day post-infection. The parasites grown in the absence of auxin showed normal growth on days 1 and 2, followed by the formation of plaques starting on day 3 (Fig. 4A). However, for parasites that were cultured in the presence of auxin, we observed unhealthy vacuoles starting on day 3 post-infection. Importantly, on day 4, the vacuoles appear to be filled with non-viable parasites with distorted morphology (Fig. 4B). These findings suggest a lack of TgAP2X-7 results in death of intracellular parasites, and hence this could be the reason for abrogation of plaque formation by parasites deficient in TgAP2X-7.

### Absence of TgAP2X-7 leads to defects in cell division

**Fig 5:**
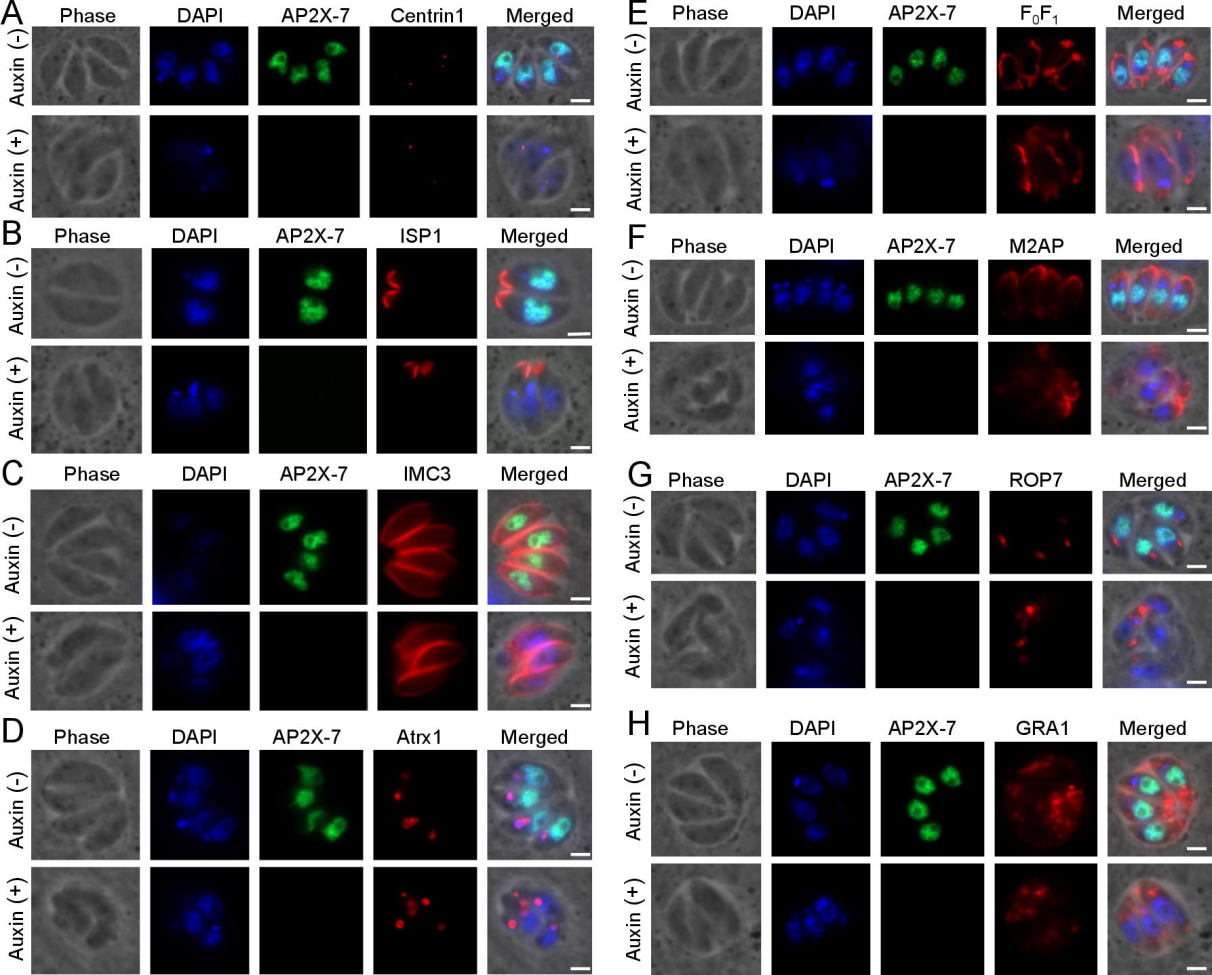
IFA analysis of TgAP2X-7.mAID.HA strain in the presence or absence of auxin using antibodies against the HA epitope and different organellar proteins. The different markers tested include (**A**) Centrin 1 as the marker for the centrosome, (**B and C**) ISP1 and IMC3 as markers for inner membrane complex (IMC), (**D**) ATRX1 as a marker for the apicoplast, (**E**) F0F1 ATPase as a marker for the mitochondrion, (**F**) M2AP as a marker for the micronemes, (**G**) ROP7 as a marker for rhoptries, and (**H**) GRA1 as a marker for dense granules. Scale bar, 2 µm.

We further wanted to determine the status of different organelles during endodyogeny in the absence of TgAP2X-7. We treated intracellular parasites with either vehicle control or auxin for 6 h and performed IFA analysis using antibodies against different parasite organellar proteins, including IMC, apicoplast, mitochondrion, micronemes, rhoptries, and dense granules (Fig. 5). We observed that while vehicle control treated parasites showed normal division with distinct daughter cells, auxin-treated parasites showed abnormal morphology (Fig. 5A through H, top panels). Specifically, the parasites were tightly packed and sometimes appeared like a mass and clearly lacked distinct organization within the vacuole (Fig. 5A through H, bottom panels). With regard to Centrin1 and ISP1, some parasites in the vacuole lacked the staining for the proteins (Fig. 5A and B). Staining with IMC3 showed that the misshapen daughter parasites are tightly opposed to each other (Fig. 5C). Loss of the transcription factor TgAP2X-7 also showed defects in plastid division with a discrepancy between the number of parasites and the apicoplast number in a vacuole (Fig. 5D). Slightly deviant morphology of the mitochondria was also evident in TgAP2X-7 depleted parasites (Fig. 5E). Further TgAP2X-7 deficient parasites showed abnormal localization of micronemal proteins (Fig. 5F) and defects in rhoptry segregation (Fig. 5G). Parasites treated with vehicle control showed the presence of dense granule proteins largely in the vacuolar space, as expected; in the auxin-treated parasites, the staining was less apparent around the parasites (Fig. 5H). Together, these findings suggest that loss of TgAP2X-7 results in defects in cell division that might be further contributing to decreased parasite viability with successive rounds of cell divisions.

### TgAP2X-7-deficient parasites show altered transcriptome

**Fig 6:**
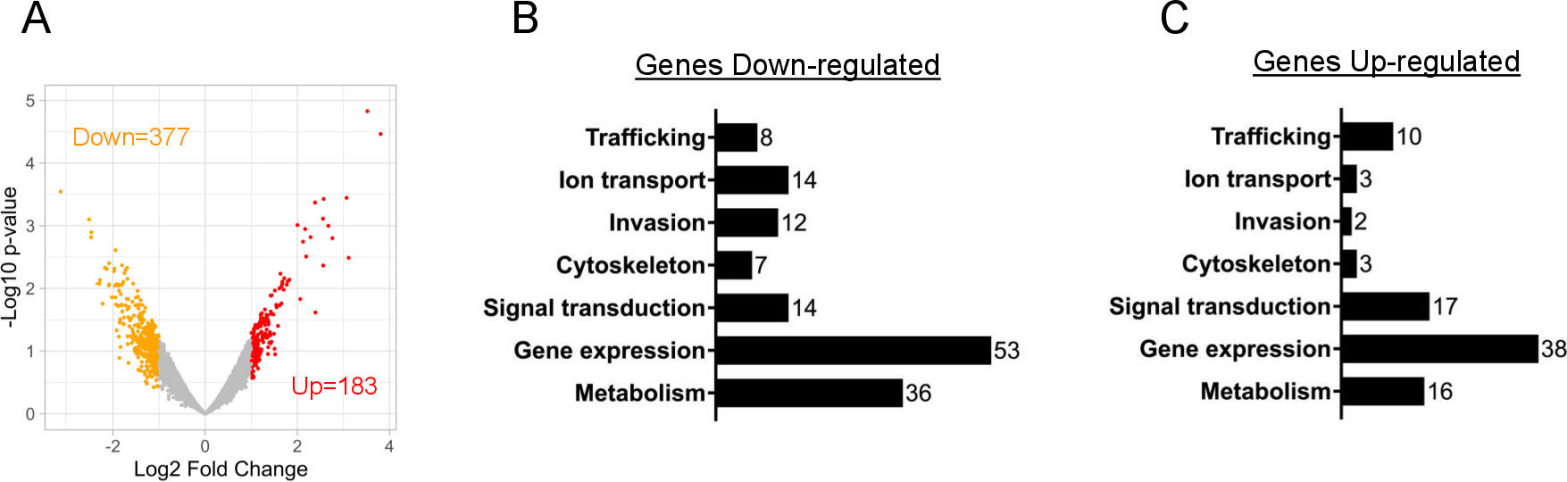
TgAP2X-7-deficient parasites show altered transcriptome. (**A**) Volcano plot of statistical significance (−Log10 *P* value) versus fold-change (log1.0), highlighting genes identified as differentially expressed (log_2_FC ≥ 1) in TgAP2X-7-mAID-HA strain treated with auxin as compared to untreated parasites. Downregulated genes are shown as orange, upregulated genes in red, and neutral genes in gray. (**B and C**) Functional classifications of genes downregulated (**B**) and upregulated (**C**) in TgAP2X-7 deficient strain compared to wild-type parasites. Classifications were manually assigned according to known gene functions or putative functions based on conserved domains. The number of genes is indicated for each functional category in both graphs.

Since TgAP2X-7 is an ApiAP2 family transcription factor, we wanted to determine if loss of TgAP2X-7 results in changes in *Toxoplasma* gene expression profile. To test this, we purified RNA from TgAP2X-7.mAID.HA strain grown with or without auxin (4 h treatment) and performed transcriptomics analysis. The RNA sequencing studies showed that there were 560 genes that were differentially expressed (log_2_FC ≥ 1) in the TgAP2X-7-deficient parasites as compared to the wild-type strain (Fig. 6A). Of these 560 differentially expressed genes, 377 genes were downregulated in parasites lacking TgAP2X-7, while 183 genes were upregulated (Fig. 6A; Data Set S1). We next manually assigned functional and cell cycle classifications for the 560 differentially expressed genes based on their known or putative functions (Fig. 6B and C; Data Set S1). Specifically, we were able to assign functional classifications to 144 of the 377 genes that were downregulated and 89 of the 183 genes that were upregulated in the absence of TgAP2X-7. The functional categorization revealed that the differentially regulated genes are associated with different functions, including gene expression, metabolism, signaling, ion transport, host-cell invasion, protein trafficking, and parasite cytoskeleton (Fig. 6B and C; Data Set S1). Interestingly, the downregulated gene set contained a higher number of invasion, metabolism, gene expression, cytoskeletal, and ion transport-related genes in comparison to the upregulated data set (Fig. 6B and C; Data Set S1). Since TgAP2X-7 shows stage-specific expression, we also examined the cell cycle status of the dysregulated gene set. Of the 377 genes that showed decreased expression, 119 genes are cell cycle regulated, 148 are constitutively expressed genes, and there is data available for the remaining genes. Within these cell cycle-regulated genes (downregulated genes), a large percentage (52%) is expressed in the G1 stage of the cell cycle (Fig. S2A; Data Set S1). Of the downregulated genes, 84 genes show cell cycle-based expression, 70 genes are constitutively expressed, and no data is available for the remainder gene set. Within the cell cycle-regulated gene set (upregulated genes), a major percentage are confined to expression in M/C phases (37%), closely followed by G1 stage (32%) (Fig. S2B; Data Set S1). Together, these findings suggest that loss of TgAP2X-7 results in global changes in *Toxoplasma* gene expression profile and specifically, TgAP2X-7 appears to be an important transcription factor involved in regulating the expression of G1 stage genes in the parasite.

### TgAP2X-7 likely binds to [C/T/G]GCATGCA[G/C/A][C/T/G][G/A] motif in the *Toxoplasma* promoter regions

**Fig 7:**
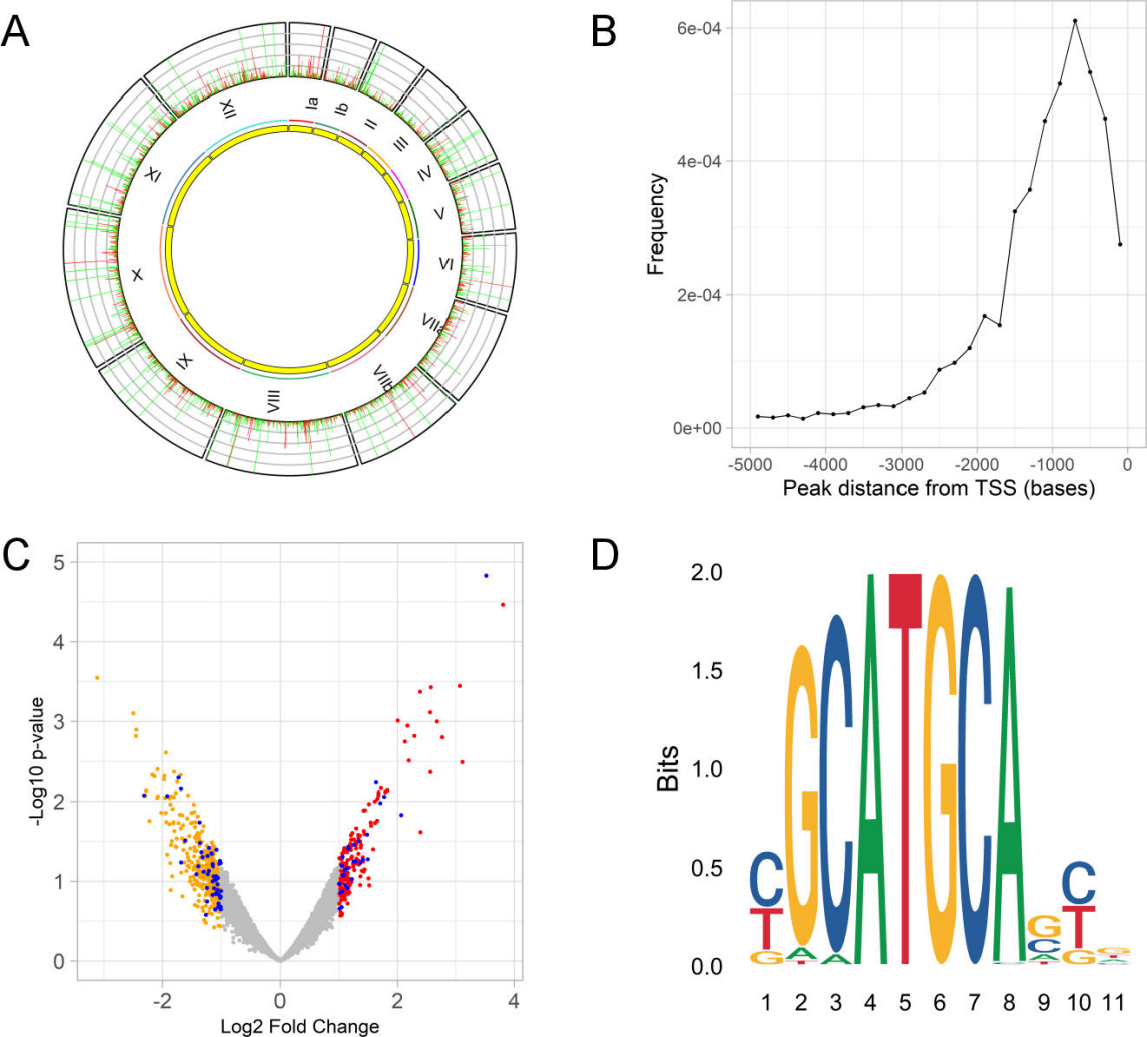
Identification of putative DNA motif recognized by TgAP2X-7 using cut and tag analysis. (**A**) Graph showing TgAP2X-7 binding peaks for the complete genome of *Toxoplasma*. The outer track shows the genomic location of CUT&TAG peaks. Radial line length corresponds to −log10 q-value, ranging from 1.3 (q-value = 0.05) to 50. For visualization, −log10 q-values above 50 were truncated to 50. Peaks within 1 kb upstream of TSS are shown in red, with the rest in green. The chromosome numbers are shown below the outer circle. (**B**) Graph showing the distribution of distances from the nearest TSS for each peak. (**C**) Genes that have TgAP2X-7 binding sites (CUT&TAG peaks) within 1 kb upstream of TSS are indicated in the volcano plot of genes dysregulated in the absence of the transcription factor. Downregulated genes are shown as orange, upregulated genes in red, and neutral in gray. Up/down-regulated genes with cut and tag peaks within 1 kb upstream of TSS are shown in blue. (**D**) CUT&TAG peaks of TgAP2X-7 are enriched for the 11 bp motif shown.

TgAP2X-7 is a member of the AP2 family proteins that contains three AP2 domains. Previous studies have shown that the AP2 domains mediate binding to specific DNA motifs (36). Since TgAP2X-7 contains AP2 domains, we wanted to determine the DNA element recognized by this transcription factor. Toward this objective, we performed CUT&TAG analysis (37, 38) using TgAP2X-7.HA strain. The CUT&TAG analysis identified a total of 3,416 peaks (Fig. 7A; Data Set S2), of which 1,398 (41%) were within 1 kb upstream of gene start sites (translational start site or TSS) of genes, which is likely to contain the promoter region (Fig. 7B; Data Set S3). Furthermore, we examined the data set of genes dysregulated in the absence of TgAP2X-7 that were identified to contain the TgAP2X-7 binding sites (peaks) in the CUT&TAG assay. The results showed that there were a total of 79 genes (50 genes in the downregulated set and 29 genes in the upregulated set) that contained AP2X-7 binding sites within 1 kb upstream of TSS (Fig. 7C), thus suggesting that these genes could be directly regulated by TgAP2X-7. Of the 50 genes that were downregulated and contain TgAP2X-7 binding peak, 27 show cell cycle regulation, 17 are constitutively expressed, while no data is available for six genes (Data Set S3). Within the cell cycle-regulated genes (downregulated and containing TgAP2X-7 binding peak), a large fraction (48%) is expressed in the G1 stage of the cell cycle (Fig. S3A). Of the 21 genes that are upregulated and have TgAP2X-7 binding peak, 16 exhibit cell cycle regulation, 10 are constitutively expressed, and no data is available for the remaining three genes (Data Set S3). For this set (upregulated and have TgAP2X-7 binding peak), a major percentage is expressed in the M/C phase (44%), closely followed by the G1 stage (31%) (Fig. S3B). The putative binding sites were found to be enriched for a single 11 bp motif ([C/T/G]GCATGCA[G/C/A][C/T/G][G/A]) with an e-value = 5.7e-005 (Fig. 7D). Together, all these findings suggest that TgAP2X-7 could be involved in regulating the expression of G1 stage genes through its interaction with the 11 bp DNA motif. However, since the TgAP2X-7 putative binding sites were found in both upregulated as well as downregulated genes, it is quite likely that TgAP2X-7 regulation of gene expression might be mediated through further post-translational regulation of the protein.

## DISCUSSION

AP2 family proteins have been shown to play a key role in regulating gene expression in apicomplexan parasites, including *Toxoplasma* (24). In this study, we sought to characterize TgAP2X-7, a member of the AP2 family in *Toxoplasma*. Our findings show that TgAP2X-7 is a nuclear protein that exhibits a cell cycle-regulated expression pattern. Since the CRISPR-based genome-wide screen aimed at identifying genes important for parasite fitness suggested that TgAP2X-7 could be essential for *Toxoplasma* propagation, we generated a conditional knockdown mutant version. Our findings reveal that TgAP2X-7 is indeed essential for parasite growth *in vitro*. Dissection of the lytic cycle biology of *Toxoplasma* using the mutant strain revealed that loss of TgAP2X-7 results in severe defects in host-cell invasion and moderate defects in the replication abilities of the parasite. Transcriptomic analysis showed that TgAP2X-7 governs a large number of genes, including those important for parasite invasion. Furthermore, CUT&TAG analysis revealed that TgAP2X-7 modulates gene expression in the parasite, most likely through binding to an 11 bp DNA motif found on *Toxoplasma* gene promoter regions.

Of the 67 APiAP2 family proteins in *Toxoplasma*, 32 have been predicted to be cell cycle regulated based on their transcriptomic profile (23, 28). TgAP2X-7 is also a cell cycle-regulated transcription factor that shows peak expression in G1, weak expression in late M/C phases, but is completely absent in S and early M/C phases. It is quite likely that expression of TgAP2X-7 is controlled at the transcriptional level; however, the cis-acting elements involved in stage-specific expression of this nuclear protein remain undetermined. Future studies focused on dissecting the TgAP2X-7 promoter would throw more light on the DNA motifs and transcription factors involved in this process.

Our findings that TgAP2X-7 is essential for parasite growth fit well with the negative phenotypic score assigned to this gene in the CRISPR-based genome-wide screen (29). Parasites deficient in TgAP2X-7 fail to form any plaques in *in vitro* growth assays. Although loss of TgAP2X-7 results in a severe defect in invasion and a moderate defect in replication, it was puzzling to see a complete loss of plaque-forming ability with parasites lacking TgAP2X-7. This prompted us to examine the day-to-day growth of these parasites post-auxin treatment, and our findings revealed that TgAP2X-7 parasites show delayed death within the vacuoles about four days post-infection. Although this now explains the abrogation of *in vitro* growth, the precise mechanism involved in the death of TgAP2X-7-deficient parasites was unclear. Thus, when we examined the events during endodyogeny, loss of TgAP2X-7 showed defects in cell division, including disorganization of daughter parasites within the vacuole, and these events could be contributing to the decreased viability and subsequent death of parasites lacking TgAP2X-7.

Transcriptomic analysis did reveal that TgAP2X-7 is involved in the regulation of gene expression in *Toxoplasma*. Interestingly, loss of this protein results in downregulation of many invasion-related genes that are important for G1 stage parasites to gain entry into the host cell. Hence, this provides a reasonable explanation for parasites showing decreased invasion ability in the absence of TgAP2X-7. Additionally, we also see that a substantial number of metabolic genes are upregulated in its absence. It is feasible that TgAP2X-7 regulates a signaling pathway that suppresses the expression of a certain set of genes, and the lack of this transcription factor allows their expression in the S and M/C phases. This might be contributing to the defects we see during replication and endodyogeny in parasites lacking this protein.

The AP2 domains in APiAP2 family proteins are about 60 amino acids in length, and these have been shown to interact with DNA motifs in the promoter regions (27, 36). TgAP2X-7 contains three AP2 domains, and our findings show that this transcription factor likely binds to [C/T/G]GCATGCA[G/C/A][C/T/G][G/A] motif. Interestingly, previous studies have shown that the first 8 bp of this motif is overrepresented in promoter regions of genes showing peak expression during the G1 stage of the cell cycle in *Toxoplasma* (31). Furthermore, the first 8 bp of this motif is similar to the TRP2 (*Toxoplasma* Ribosomal Protein 2) cis-element that was shown to be required for transcription of ribosomal protein genes in the parasite that are also upregulated in the G1 stage (39, 40). Furthermore, the putative TgAP2X-7 binding motif shares significant homology with the binding site of the transcription factor, TgAP2XII-8, that was shown to be involved in progression through G1 phase of the cell cycle (41). Together, these findings suggest TgAP2X-7 is another major transcription factor that plays a key role in the modulation of genes showing peak expression in the G1 stage of the cell cycle. Additionally, this motif is also present in a small number of genes (some of which have peak expression in the S and M/C phases) that are upregulated in the absence of TgAP2X-7. These observations also imply that TgAP2X-7 might be subjected to additional levels of regulation in its functioning. This could be either through post-transcriptional modifications, such as phosphorylation by nuclear kinases or interaction with other transcription factors or chromatin modifiers. Previous studies have shown that TgAP2X-7 is phosphorylated at multiple sites, and it is feasible that one or more of these events could be involved in modulating TgAP2X-7 function (42, 43). Hence, more advanced studies focused on identifying the interactome of TgAP2X-7, as well as mutational analysis of these phosphorylated residues, will reveal the signaling pathway governing the function of this novel transcription factor.

## MATERIALS AND METHODS

### Parasite cultures

*Toxoplasma gondii* tachyzoites were maintained by passage through HFF in a humidified incubator at 37°C with 5% CO_2_. Normal growth medium consisted of DMEM supplemented with 10% fetal bovine serum, 2 mM L-glutamine, and 50 µg/mL of penicillin-streptomycin. Purification of parasites was performed as previously described (44).

### Endogenous tagging of TgAP2X-7 with HA and mAID.HA epitope tags

The endogenous tagging of TgAP2X-7 at the C-terminus was performed according to previously published protocols (32, 45, 46). Briefly, a single guide RNA (sgRNA) plasmid containing a protospacer against the 3′ untranslated region (UTR) of TgAP2X-7 downstream of the stop codon was generated through site-directed mutagenesis. The homology-directed repair (HDR) templates were PCR amplified using the vectors, p3XHA.LIC-DHFR and pmAID3xHA.LIC-HPT that contain HA and mAID.HA epitope tags, respectively. Additionally, both plasmids contain a selection cassette that provides resistance against pyrimethamine (DHFR) and MPA-Xanthine (HXGPRT) (47, 48). The 60 bp primers used in generating the repair template include 40 bp of homology immediately upstream of the stop codon or 40 bp of homology within the 3′ UTR downstream of the CRISPR/Cas9 cut site. All primers that were used for pU6-Universal plasmids and HDR templates are listed in Table S1. The sgRNA plasmid and the HDR templates were then transfected into the RHΔKu80 strain using nucleofector (49). Transfected parasites were then cultured in the presence of either pyrimethamine or MPA/xanthine to select stably transformed parasites that were cloned by limiting dilution. The clones were screened and validated by PCR and sequencing.

### Immunofluorescence microscopy

Immunofluorescence staining of intracellular parasites was performed according to a previously described procedure (50). The primary antibodies used were mouse anti-HA (Cell Signaling Technology, 6E2, 1:1,000), rabbit anti-HA (Cell Signaling Technology, C29F4, 1:1,000), rabbit anti-IMC3 (1:2,000) (51), rabbit anti-centrin 1 (52), and mouse anti-ISP1 (1:1,000) (53). Secondary antibodies used include Alexa Fluor-594- or Alexa Fluor-488-conjugated goat anti-rabbit or goat anti-mouse (Molecular Probes, 1:1,000). Slides were viewed using a Zeiss Axio Observer 7 microscope (Carl Zeiss), and digital images were captured with an Axiocam 506 mono charge-coupled device camera using Axiovision software.

### Immunoblotting assay

Immunoblotting assays were performed according to previously published protocols (50). The primary antibodies used were anti-HA rabbit mAb C29F4 (1:2,500; Cell Signaling Technology) and anti-*T*. *gondii* SAG1 (1:10,000). Secondary antibodies used were goat anti-rabbit and goat anti-rat IgG conjugated to horseradish peroxidase (Jackson Immuno Research Laboratories, West Grove, PA). After washing, membranes were treated with SuperSignal West Femto chemiluminescent substrate (Pierce Chemical) and imaged using Flourchem Protein Simple.

### Plaque assays

Plaque assays were performed as described previously with some modifications (54, 55). Intracellular parasites were harvested, syringe filtered, and added onto a confluent monolayer of HFF cells in a 12-well plate (500 tachyzoites per well). The plates were then incubated at 37°C for 6 days without any movement. The plates were then washed with PBS, methanol fixed, and stained with 2% crystal violet to visualize regions of host cell disruption.

### Invasion assay

Invasion assays were performed in eight-well chamber slides as described previously with the following modifications (35, 56). Briefly, parasites were pretreated with vehicle control or 500 µM indole-3-acetic acid (IAA) (to deplete the mAID-3HA tagged TgAP2X-7) in DMEM with 10% FBS for 4 h. Purified tachyzoites were then added onto HFF monolayers (2 × 10^6^ parasites per well) and incubated at 37°C for 30 min in the presence or absence of auxin. Slides were then washed three times to remove non-invaded parasites, fixed, blocked, and stained with mouse anti-SAG1 without permeabilization. After 1 h, slides were washed, permeabilized with 0.01% TX-100, and stained with rabbit anti-M2AP antibody. The slides were further washed and stained with secondary antibodies, Alexa Fluor-594-conjugated goat anti-mouse (Molecular Probes) and Alexa Fluor-488-conjugated goat anti-rabbit (Molecular Probes). After 1 h, slides were washed and mounted using Vectashield (with DAPI). Parasites that were both red and green were identified as extracellular (attached), whereas those that were green but not red were identified as intracellular (invaded). Images of 15 random fields of view within each well were captured at 600× magnification, and the total number of intracellular parasites and host cell nuclei was enumerated.

### Replication assay

To assess the parasite doubling time, freshly egressed parasites were inoculated into confluent HFF monolayers in 12-well plates and allowed to invade for 2 h. The monolayers were then washed three times with medium to remove uninvaded parasites and incubated at 37°C in DMEM with 10% FBS containing vehicle control or the presence of 500 µM IAA. At 30 h post-infection, the cells were fixed with methanol and stained using Diff-Quik (Dade-Behring) according to the manufacturer’s instructions. For each treatment, at least 100 vacuoles from three biological replicates were assessed for the number of parasites per vacuole.

### Ionophore-induced egress assay

The efficiency of egress after calcium ionophore treatment was determined using established protocols (57). Briefly, freshly harvested parasites were added to a 24-well plate containing confluent HFFs at a multiplicity of infection of 1 and were incubated at 37°C. After incubation for 27 h, the cultures were treated with 500 µM IAA or vehicle control for 4 h to induce degradation of TgAP2X-7.mAID.HA protein. Then, to induce egress, intracellular parasites were washed with warm PBS, incubated at 37°C for 2 min in Hanks' balanced salt solution containing 1 µM calcium ionophore A23187, and fixed with 100% methanol. To visualize intact and lysed vacuoles, the cultures were stained using Diff-Quik (Dade-Behring) according to the manufacturer’s instructions. Percent egress was determined by dividing the number of lysed vacuoles by the total number of vacuoles for a sample.

### Endodyogeny assays

TgAP2X-7.mAID.HA parasites were added onto cover slips with a confluent host cell monolayer and incubated at 37°C. Twelve hours post-incubation, coverslips were washed and incubated with DMEM with 10% FBS containing vehicle control or 500 µM IAA for 6 h. The coverslips were then washed, fixed, and stained with antibodies against HA epitope mouse anti-HA (Cell Signaling Technology, 6E2, 1:1,000), rabbit anti-HA (Cell Signaling Technology, C29F4, 1:1,000) and different organellar marker proteins, including rabbit anti-IMC3 (1:2,000) (53), rabbit anti-centrin 1 (52), mouse anti-ISP1 (1:1,000) (58), rabbit anti-(Apicoplast Thioredoxin 1) ATRX1 (1:500) (59), rabbit anti-F_0_F_1_ ATPase (1:1,000) (60), rabbit anti-M2AP (1:1,000) (35), rabbit anti-ROP7 (1:500) (61) and rabbit anti-GRA1 (1:500) (62). Secondary antibodies used include Alexa Fluor-594- or Alexa Fluor-488-conjugated goat anti-rabbit or goat anti-mouse (Molecular Probes, 1:1,000). Slides were viewed using a Zeiss Axio Observer 7 microscope (Carl Zeiss), and digital images were captured with an Axiocam 506 mono charge-coupled device camera using Axiovision software.

### RNA sequencing and differential gene expression analysis

RNA sequencing was performed according to previously published protocols with some modifications (49, 63). Total RNA from TgAP2X-7.mAID.HA parasites treated with 500 µM IAA or vehicle control for 4 h was isolated using the RNeasy kit (Qiagen). RNA samples were obtained from three independent experiments. The quality of total RNA samples was verified by using a BioAnalyzer (Agilent), followed by digestion with DNase I (NEB). Ribosomal RNA was removed using the Ribo-Zero rRNA removal kit (human/mouse/rat, Illumina). Sequencing libraries were then generated using the TruSeq RNA Sample Prep Kit (v2, Illumina) according to the manufacturer’s protocol. Libraries were amplified using the TruSeq Cluster Kit (v3, Illumina) and subjected to 50 bp single-end sequencing with the Illumina HiSeq 2000 system. Sequencing reads were aligned to the *Toxoplasma* GT1 reference genome (ToxoDB v.53, https://toxodb.org/toxo/app) using the STAR software package (v.2.7.1a, with default settings) (64). Filtered and normalized gene expression levels were calculated from the aligned reads using HTSeq v.0.13.5 (65). Differentially expressed genes were identified by linear modeling and Bayesian statistics using the limma package for R v.3.49.1 (66).

### Cleavage under targets and tagmentation assay, and sequencing analysis

The analysis of chromatin occupancy for TgAP2X-7.mAID.HA protein was performed by CUT&TAG assay using CUT&TAG-IT Assay Kit (Cat no: 53160, anti-rabbit) from Active Motif according to the manufacturer’s instructions with some modifications (38). Samples from three independent experiments were used in the analysis. Briefly, TgAP2X-7.HA tachyzoites were harvested and syringe filtered. The purified tachyzoites (2 × 10^7^) were then pelleted at 1,000 × *g* for 10  min at room temperature. The parasite pellet was then washed, followed by binding to Concanavalin A beads. The bound parasite cells were then treated with rabbit anti-HA antibody, followed by guinea pig anti-rabbit secondary antibody (in the negative control, only the rabbit anti-HA antibody was excluded, while the rest of the procedures were performed as described). This was followed by assembly of pA-Tn5 transposomes, tagmentation, and DNA extraction. The PCR reactions were performed using i7 indexed primers provided in the kit, followed by SPRI bead clean-up. The DNA libraries were then generated and sequenced using the Illumina sequencing platform (PE, 250 bp). The sequencing reads were mapped to the *T. gondii* reference genome (TgondiiGT1-53) using the NVIDIA Parabricks implementation of the Burroughs Wheeler Aligner (PB-BWA version 4.3.0-1) (67, 68). Mapping quality was assessed using samtools version 1.3.1 (69). Possible binding regions (peak regions) were identified using Model-based Analysis of ChIP-seq (MACS version 2.2.7.1) (70). Possible binding motifs based on peak region sequences were determined using STREME (71) within MEME-chip Suite Version 5.5.6 (72).

## Data Availability

The RNAseq and CUT&TAG data generated in this study have been deposited in the Sequence Read Archive (SRA) under the BioProject ID PRJNA1281009.

## SUPPLEMENTAL MATERIAL

Data Set S1 msphere.00438-25-s0001.xlsx RNAseq data.

Data Set S2 msphere.00438-25-s0002.xlsx CUT&TAG analysis data.

Data Set S3 msphere.00438-25-s0003.xlsx Direct targets of TgAP2X-7.

Figure S1 msphere.00438-25-s0004.tiff TgAP2X-7 is a cell cycle-regulated transcription factor.

Figure S2 msphere.00438-25-s0005.tiff Loss of TgAP2X-7 results in dysregulation of cell cycle regulated genes.

Figure S3 msphere.00438-25-s0006.tiff Cell cycle classification of putative target genes of TgAP2X-7.

Legends msphere.00438-25-s0007.pdf Supplemental material legends.

Table S1 msphere.00438-25-s0008.pdf Primers.

ASM does not own the copyrights to Supplemental Material that may be linked to, or accessed through, an article. The authors have granted ASM a non-exclusive, world-wide license to publish the Supplemental Material files. Please contact the corresponding author directly for reuse.
