# Supplementary figures and images for: TgAP2X-7 is a novel cell cycle-regulated transcription factor that plays an essential role in Toxoplasma tachyzoite propagation
Source: mSphere. 2025 Sep 8;10(9):e00438-25. doi: 10.1128/msphere.00438-25 (PMC12482157; doi:10.1128/msphere.00438-25)

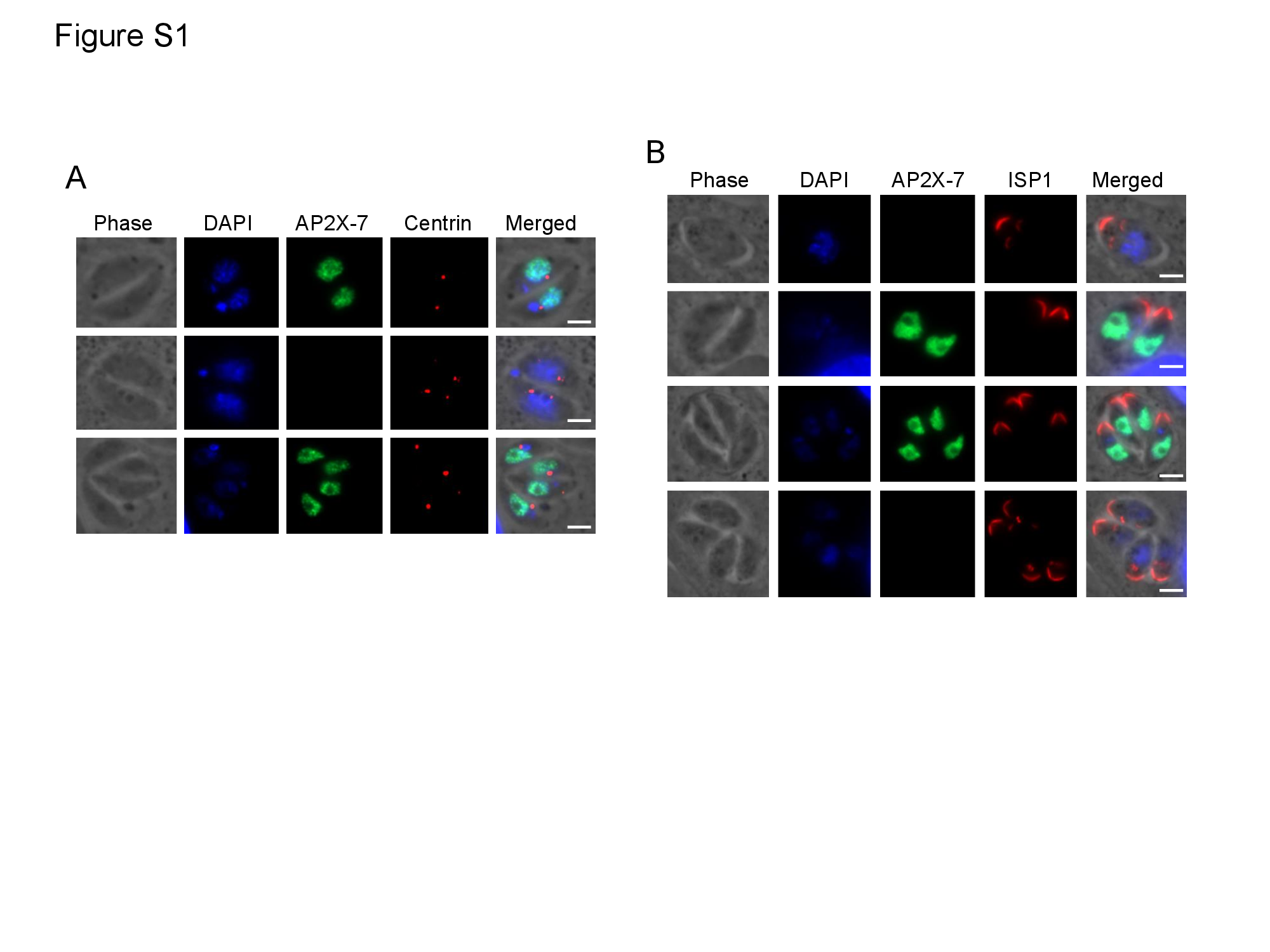

Supplement: Figure S1 — TgAP2X-7 is a cell cycle-regulated transcription factor. [file msphere.00438-25-s0004.tiff]

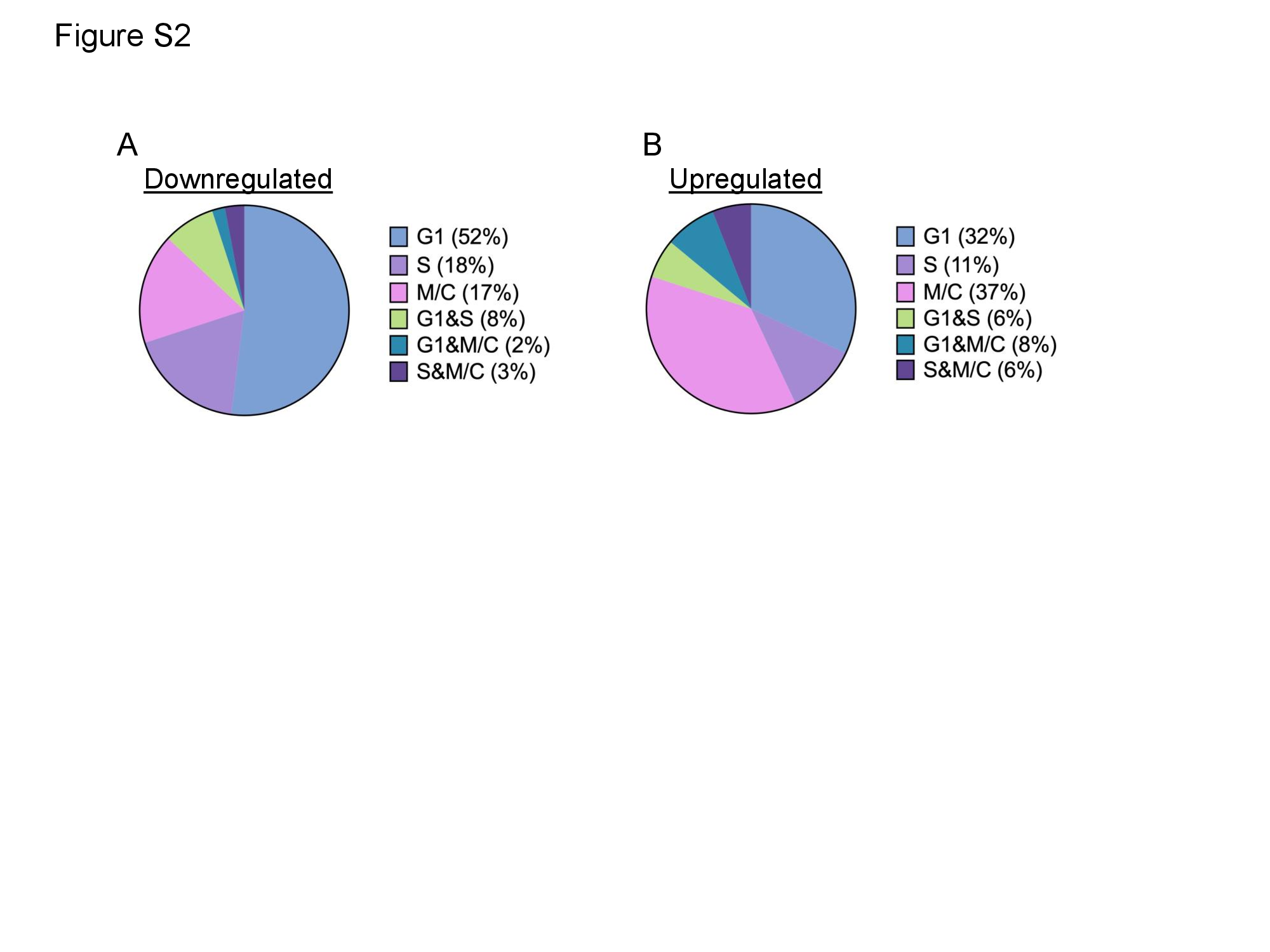

Supplement: Figure S2 — Loss of TgAP2X-7 results in dysregulation of cell cycle regulated genes. [file msphere.00438-25-s0005.tiff]

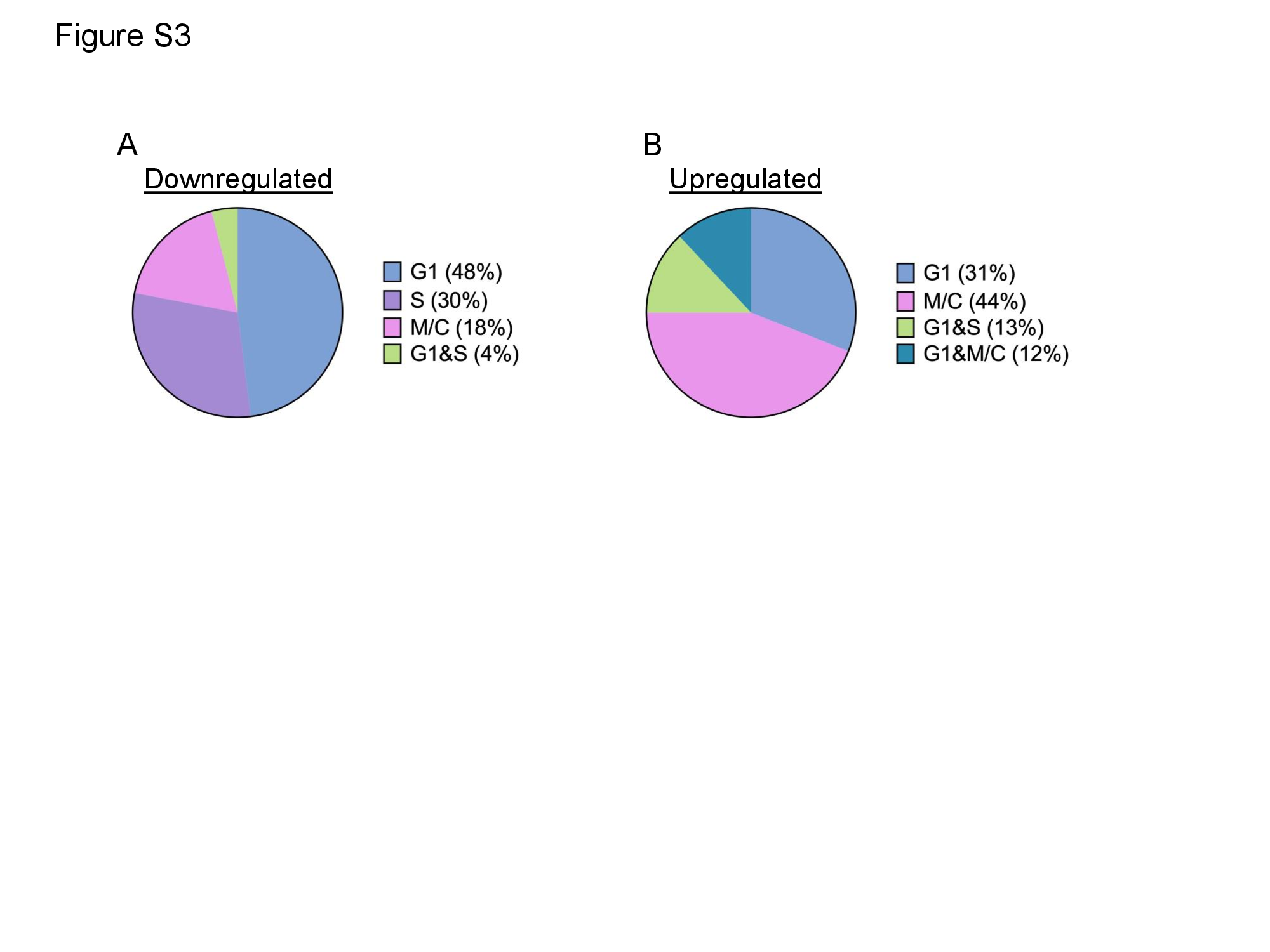

Supplement: Figure S3 — Cell cycle classification of putative target genes of TgAP2X-7. [file msphere.00438-25-s0006.tiff]
