# Supplementary material for: TgAP2X-7 is a novel cell cycle-regulated transcription factor that plays an essential role in Toxoplasma tachyzoite propagation
Source: mSphere. 2025 Sep 8;10(9):e00438-25. doi: 10.1128/msphere.00438-25 (PMC12482157; doi:10.1128/msphere.00438-25)
Supplement: Legends — Supplemental material legends. [file msphere.00438-25-s0007.pdf]

Legends for supplemental material

LEGENDS FOR SUPPLEMENTAL FIGURES

Figure S1. TgAP2X-7 is a cell cycle regulated transcription factor. Localization of TgAP2X-7 in intracellular parasites at different stages of cell cycle using anti-HA antibody. Centrin1 is used as a marker for centrosome and ISP1 is used as a marker for early daughter cell budding. DAPI stains the nucleus. Scale bar, 2 μm.

Figure S2. Loss of TgAP2X-7 results in dysregulation of cell cycle regulated genes. Pie charts showing cell cycle classification of genes (A) downregulated and (B) upregulated in the absence of TgAP2X-7. The percentages are rounded to the nearest whole number.

Figure S3. Cell cycle classification of putative target genes of TgAP2X-7. Pie charts showing cell cycle classification of genes (A) downregulated and (B) upregulated in the absence of TgAP2X-7 and contain TgAP2X-7 binding peak. The percentages are rounded to the nearest whole number.

SUPPLEMENTAL DATA LEGENDS

1. Supplemental table S1. List of primers used in this study.

2. Supplemental data set S1. List of genes dysregulated in TgAP2X-7 conditional knockdown mutant strain.

3. Supplemental data set S2. List of TgAP2X-7 binding peaks in the CUT&TAG analysis along with binding site location on the chromosome.

4. Supplemental data set S3. List of CUT&TAG peaks with distances to nearest upstream transcription start site. The cell cycle expression information for the TgAP2X-7 target genes is also included.
