# Supplementary material for: TgAP2X-7 is a novel cell cycle-regulated transcription factor that plays an essential role in Toxoplasma tachyzoite propagation
Source: mSphere. 2025 Sep 8;10(9):e00438-25. doi: 10.1128/msphere.00438-25 (PMC12482157; doi:10.1128/msphere.00438-25)
Supplement: Table S1 — Primers. [file msphere.00438-25-s0008.pdf]

Table S1. Primers used in this study.

S. No	Name	Sequence
1	AP2X7.sgRNA.F	gttacagttcGTTTTAGAGCTAGAAATAGC
2	AP2X7.sgRNA.R	tgttgtttgcAACTTGACATCCCCATTTAC
3	AP2X7.HA.RT.F	AGAGAAGCCAGAGGAGAATGCAGCGCCCAGTGAGGAGCAGGGAAGTGGAGGACGGGAATT
4	AP2X7.HA.RT.R	CGTGAATCAGAATTTCAGATCTCCTGTGCATCTGCTTCCGACGGCCAGTGAATTGTAATA
5	AP2X7.mAID.RT.F	AGAGAAGCCAGAGGAGAATGCAGCGCCCAGTGAGGAGCAGgagctgtacaagcctaggat
6	AP2X7.mAID.RT.R	AGATCTCCTGTGCATCTGCTTCTCGAAACGTTGCACGGAAcgctctagactgcagataac
